# Tuberculin Dispensaries

**Published:** 1913-03-01

**Authors:** W. Camac Wilkinson

**Affiliations:** Director Tuberculin Dispensary. 8 Upper Wimpole Street, W.


### Tuberculin Dispensaries.

*To the Editor of* The Hospital.

Sir,—You do not seem to be aware that ambulant treatment of tuberculosis by means of tuberculin has been practised at our tuberculin dispensary for the last three years and Dr. Morland paid us a visit. If you have time you might find it useful to pay one visit, or, still better, many visits, so that you may understand what we are doing. Perhaps then you might not attach the same importance to a visit from Dr. Morland who has not himself used tuberculin in any comprehensive way, although he has translated Bandelier and Roepki's work upon tuberculin treatment.

It is remarkable that so many writers about tuberculin would rather trust to hearsay evidence than take the trouble to investigate direct evidence which lies at their very door.

If you come to the dispensary I shall do all in my power to show you our methods of administration.—Yours faithfully,

W. Camac Wilkinson,
Director Tuberculin Dispensary.

8 Upper Wimpole Street, W.,
February 24, 1913.

